# The impact of item-writing flaws and item complexity on examination item difficulty and discrimination value

**DOI:** 10.1186/s12909-016-0773-3

**Published:** 2016-09-29

**Authors:** Bonnie R. Rush, David C. Rankin, Brad J. White

**Affiliations:** Department of Clinical Sciences, College of Veterinary Medicine, Kansas State University, Manhattan, KS USA

**Keywords:** Multiple choice examinations, True-false examinations, Pre-clinical education, Item-writing flaws, Veterinary education

## Abstract

**Background:**

Failure to adhere to standard item-writing guidelines may render examination questions easier or more difficult than intended. Item complexity describes the cognitive skill level required to obtain a correct answer. Higher cognitive examination items promote critical thinking and are recommended to prepare students for clinical training. This study evaluated faculty-authored examinations to determine the impact of item-writing flaws and item complexity on the difficulty and discrimination value of examination items used to assess third year veterinary students.

**Methods:**

The impact of item-writing flaws and item complexity (cognitive level I-V) on examination item difficulty and discrimination value was evaluated on 1925 examination items prepared by clinical faculty for third year veterinary students.

**Results:**

The mean (± SE) percent correct (83.3 % ± 17.5) was consistent with target values in professional education, and the mean discrimination index (0.18 ± 0.17) was slightly lower than recommended (0.20). More than one item-writing flaw was identified in 37.3 % of questions. The most common item-writing flaws were awkward stem structure, implausible distractors, longest response is correct, and responses are series of true-false statements. Higher cognitive skills (complexity level III-IV) were required to correctly answer 38.4 % of examination items. As item complexity increased, item difficulty and discrimination values increased. The probability of writing discriminating, difficult examination items decreased when implausible distractors and all of the above were used, and increased if the distractors were comprised of a series of true/false statements. Items with four distractors were not more difficult or discriminating than items with three distractors.

**Conclusion:**

Preparation of examination questions targeting higher cognitive levels will increase the likelihood of constructing discriminating items. Use of implausible distractors to complete a five-option multiple choice question does not strengthen the discrimination value.

**Electronic supplementary material:**

The online version of this article (doi:10.1186/s12909-016-0773-3) contains supplementary material, which is available to authorized users.

## Background

The goal of examination item preparation is to develop testing methods that do not confuse students and yield scores that accurately reflect the extent to which students have obtained a satisfactory working knowledge of the content. Well-constructed multiple choice examinations represent a versatile assessment tool with the potential to assess students for sufficient working knowledge of the tested content [1–4]. Discriminating multiple-choice questions are difficult and time-consuming to prepare [5, 6]. Examination experts estimate a quality multiple choice question requires 20 to 60 min to construct and item-writing flaws are common in faculty-prepared examinations [5, 7, 8]. Item-writing flaws may render examination questions easier or more difficult than intended [7, 9–14]. Some flaws provide clues that allow unprepared students to guess the correct answer; whereas awkward, unnecessarily complex or esoteric examination items prevent prepared students from demonstrating their knowledge [7, 9].

### Items easier than intended

The following standard item-writing flaws are considered to make examination items easier than intended, favoring test-wise students [15]:

- Longest option is correct answer.
- Grammatical clues or inconsistencies between stem and distractors.*
- Implausible distractors.*
- Mutually exclusive distractors.*
- Use of absolute terms (always, never, only, all).
- Use of all of the above.

*Examples appear in Additional file 1.

The *longest option is correct answer* is a common mistake made by novice and experienced examination writers in an effort to ensure the correct response is indisputable [16]. *Grammatical clues* may come in the form of syntax inconsistencies between the stem and incorrect responses (distractors), or may occur if a key word in the stem is repeated in the correct response [16]. *Implausible distracters* are used to create item uniformity when three or four plausible distracters are immediately apparent to the author [2, 17, 18]. Students recognize *mutually exclusive distractors* and conclude that one of the two mutually-exclusive responses is correct, eliminating other options [7]. Test-savvy students also recognize that *absolute terms* (always, never) usually render a statement false.

For students to identify “*all of the above*” as the correct response, they need only identify two correct answers among the choices. To determine “all of the above” is incorrect, students need only identify one false statement [13]. High school and undergraduate students are coached to select “all of the above” on standardized examinations, unless there is clear evidence to the contrary; “all of the above” is the correct response 52 % of the time on standardized examinations [19].

Author selection for denotation of the correct response is reported to be statistically predictable. Novice examination writers under-utilize options A and E as correct responses, and overuse option C as the correct response [7]. In one report, E was identified as the correct response 5 % of the time [13]. This pattern provides a strategic advantage for experienced examinees.

### Items more difficult than intended

The following item-writing flaws may render questions unnecessarily complex and prevent prepared students from demonstrating mastery of the material: [7, 13, 16].

- Awkward stem structure. (Finish the sentence, fill in the blank, grammatically flawed.)*
- Extraneous or misleading information in the stem.*
- Negative stem. (Not true, true except, incorrect.)
- Response options are a series of true/false statements.*
- Use of none of the above.
- Complex or K-type items. (e.g. A and C)
- Vague or generalizing terms. (Sometimes, frequently, often, occasionally, typically, potentially).
- Unfocused question. (Distractors are unrelated or distantly related to a single learning objective.)*

*Examples in Additional file 1.

Item-writing experts recommend that the stem be a complete sentence and represent a stand-alone problem [7]. In other words, students should be able to formulate a projected correct response, based on the stem alone. Questions with *awkward stem structure* require students to place each response option in the blank or at the end of the sentence, which can result in an error unrelated to the learning objective. *Extraneous or misleading* information in the item stem distracts students from the learning objective, lengthens the examination unnecessarily, and decreases the reliability and the validity of the resulting test scores [16].

*Negatively-worded multiple choice stems* instruct students to identify the incorrect answer among response options. (e.g. Which of the following is not true/incorrect/false?) Negatively worded questions are easier to construct than positively worded questions, however, learning objectives are more effectively assessed when students identify a correct answer rather than an incorrect answer [11, 16]. A stem and response option combination that results in a double negative is particularly difficult for students to answer correctly. In health education, questions regarding inappropriate (contraindicated) treatment are warranted and represent an exception to this guideline. In these situations, “contraindicated” should be bolded, underlined, and appear in capital letters; each response option should be phrased positively to avoid forming a double negative with the stem [7].

One variant of multiple-choice questions requires students to evaluate response options that are essentially a *series of true/false statements*. (e.g. Which of the following statements is correct/incorrect?) Consequently, students are responding to four or five true/false questions with all-or-none grading, rather than one multiple choice item [20, 21]. When the stem is written in the negative form (false/incorrect/not true), the item violates two standard item-writing guidelines. A series of true/false responses is a popular question format because the structure allows the examiner to cover a wide range of material. Most evaluations of true/false response options indicate this format disadvantages students with knowledge of the tested content [20].

Use of “none of the above” does not require students to demonstrate knowledge of the correct answer. When used as a distractor, it typically does not appear plausible to experienced students, and becomes a filler or implausible distractor. Use of “none of the above” decreases item discrimination and test score reliability [16]. Current guidelines do not recommend use of this option, except by highly experienced item writers [7].

*K-Type questions*, also known as *complex items*, require students to select combinations of individual response options. Response options are typically a series of true/false statements, followed by options such as “A and B” or “two of the above” [22]. K-type items share the disadvantage of all-or-none scoring in a true/false format. In addition, K-type questions often provide grammatical clues that help experienced students detect the correct combination of alternatives. Two of the above is flawed because students might not select the intended correct answers, yet still receive credit. Studies indicate K-type questions perform poorly in reliability and discrimination when compared with single-correct-answer and true/false formats [7, 16, 20].

*Vague or generalizing terms* open examination questions to interpretation. The terms “frequently”, “often” “sometimes” or “occasionally” may hold different meaning to the writer and student, and the impact may vary with circumstances or disease condition [7]. An *unfocused stem* is a broad, open-ended question that does not pose a specific problem and is followed by a series of unrelated response options. This item type is popular because it allows examiners to test a broad range of material, but does not provide an assessment of a specific learning objective, and has a detrimental impact on student and item performance [7, 21].

### True/false questions

True/false questions tend to be easy to write and efficiently answered. Students can respond to approximately 50 true/false items in the time it takes to answer 30 multiple-choice items [14, 21]. Consequently, true/false examination items provide the widest sampling of content per unit of time. The primary disadvantage is guessing [22]. Students have a 50 % chance of correctly answering an item without knowledge of the material. For this reason, licensing organizations, including the National Board of Medical Examiners, have removed true/false items from their question bank [14]. False items tend to discriminate more highly than true items. When students guess, they are more inclined to respond with true than with false. Test writers are advised to compensate by offering more false statements than true [23].

### Item complexity

Examination item complexity is categorized using a five level scale based on Bloom’s Taxonomy [24, 25]: knowledge, comprehension, application, analysis, and synthesis/evaluation [1, 3, 26, 27]. Five categories (levels I through V) are grouped within two subheadings (lower and higher complexity). Lower level complexity (I and II) requires students to recall factual knowledge (I) or conceptual understanding (II). Higher level complexity (III through V) requires application of knowledge. For example, level III items may require students to determine the next diagnostic or therapeutic decision in a prototypic case example. Level IV items require students to analyze conceptual knowledge through interpretation and integration of multiple data points from clinical findings or diagnostic testing. Students are then asked to make a prediction or select a course of action [5]. Level V items test students’ ability to evaluate procedural knowledge; a case study is presented from patient presentation to conclusion and students are asked to identify an error or alternative plan within the case. Examples of examination items categorized as level I through V appear below:

Recall (lower level cognition)

I. Factual recall: Which of the following species does not have a gall bladder?
II. Conceptual recall: Which of the following conditions is an example of ventilation-perfusion mismatch?

Applied (higher level cognition)

III. Direct Application: Simple case scenario - Which of the following diagnostic tests is indicated?
IV. Analyze conceptual knowledge: Interpret blood work, radiograph, or complex case scenario.
V. Evaluate case management: Case vignette - Which steps were unnecessary?

Examination items testing lower cognitive thinking (I and II) are easier to write, yet are more prone to item-writing flaws and poor discrimination ability [13]. Challenging items that test higher cognitive thinking (III-V) require experience, creativity, and time to construct [6]. As students approach application of subject matter in a practical setting (i.e. field training, clerkship, or practicum), a greater proportion of higher level cognition items are recommended for evaluation. Higher level cognition items assess critical thinking skills, serve as an advanced learning tool, and facilitate content retention [1, 3, 26].

### Post-examination item analysis

The discrimination index indicates the extent that success on the item is related to the success on the test as a whole, and provides feedback to the examiner regarding item difficulty. The index is the difference between the percentage of correct responses from the upper and lower scores of the class, demonstrating the impact of an item to distinguish between high scorers and low scorers on an examination. Varying values are used to define upper and lower student groups, in many cases, upper and lower quartiles are used. The target value for the discrimination index should be approximately 0.20 for examination items, except intentionally easy or difficult questions [14, 28–30]. Items with low (less than 0.10) or negative indices should be reviewed to determine whether the item is flawed or mis-keyed.

### Objectives

The goals of this investigation are to determine the impact of item-writing flaws and item complexity on the difficulty and discrimination value of examination items authored by in-house veterinary faculty and administered to third year veterinary students. Most reports of item-writing flaws reflect evaluation of students in undergraduate course work or basic science curriculum [4, 6, 9, 13, 17], and include a single course or single examination [1, 3, 4]. This report represents student examinations administered over a complete academic year of professional curriculum. Third year veterinary students are experienced multiple choice examinees (7.2 years of higher education at this stage of training). All examination questions administered during the third year are authored by practicing clinical faculty. Students begin their clinical training program, supervised by these same faculty, upon successful completion of the third year of the curriculum. We hypothesize that this population of students may be less impacted by item-writing flaws than previous reports.

## Methods

### Participants

Data were collected from all examination questions administered over the academic year for third year veterinary students (112 students; 85 female, 27 male; average age = 25.9 years). At the start of the investigated academic year, third year student respondents had completed an average of 7.2 years of post-secondary education. Most veterinary students had completed a baccalaureate degree prior to matriculation. All examination questions were authored by college of veterinary medicine (CVM) faculty members, intended to have one correct response, and assessed via automated grading (Scantron™^1^). Courses included small animal medicine [8 credit hours (cr)], small animal surgery (5 cr), large animal medicine (7 cr), large animal surgery (4 cr), nutrition (2 cr), reproduction (3 cr), zoological medicine (2), ethics/jurisprudence (1 cr), clinical pharmacology (2 cr) and practice management (1 cr). This study was determined by the Institutional Review Board administrator of Kansas State University to be exempt from full panel review.

### Procedures

Objective data was documented for each examination item including author, course, elective/core, correct response (e.g. A, B, C, D, E), question number (order of examination questions), length of stem, length of responses, use of ancillary materials (photographic image, radiographic image, video, line drawing), interpretation of laboratory values, and calculation requirement (yes/no).

Two item raters (BRR and DCR) evaluated each examination item independently for item-writing flaws and item complexity. When disagreement was observed between raters, raters discussed and reached consensus for each examination item. Raters had content-area expertise, experience preparing multiple choice items, and NBME item-writing training.

Raters evaluated examination items for case-based question format (yes/no) and the presence of the following item-writing flaws: longest response is correct, grammatical clues, implausible distractors, mutually exclusive distractors, use of absolute terms, use of all of the above, awkward stem, misleading/extraneous stem, negative stem, true/false distractors, use of none of the above, K-type responses, use of vague terms, and unfocused question.

A rating of item complexity (cognitive level I-V) was assigned by the raters for each question based on modified Bloom’s taxonomy [1, 3, 26]. Item performance (psychometric) parameters were collected from reported examination statistics including percent correct, selection of distractors, discrimination index, and examination difficulty (class average on the examination in which the item was used). At Kansas State University, the discrimination index is reported for each item using responses from the upper 27 % and lower 27 % students, categorized by their performance on the entire examination.

### Analysis

Data were prepared for analysis by removing questions where more than one response option was deemed correct (20 questions, nine instructors), questions in which no correct answer was identified (five questions, one instructor), and one question from an instructor with only a single question in the data set.

Main outcome variables (% correct and discrimination index) were log-transformed to normalize distributions. Categorical variables evaluated questions that were difficult with poor discrimination (<70 % correct; discrimination index <0.15), easy with poor discrimination (>90 % correct; discrimination index <0.15), and challenging with strong discrimination (< 85 % correct and index >0.20). Stepwise regression models were created to evaluate relationships among the percent correct, index values, and categorical description of discriminatory value of question compared with all available variables including course, course type (elective/core), test number within course, instructor, stem length, distractor length, case-based (yes/no), complexity, ancillary (yes/no), and the standard item-writing flaws: longest response is correct, grammatical clues, implausible distractor, mutually exclusive distractors, use of absolute terms, use of all of the above, awkward stem, misleading/extraneous stem, negative stem, true/false distractors, use of none of the above, k-type responses, use of vague terms, and unfocused question. To account for lack of independence of questions, course, test number within course, and instructor were forced into all models. Multivariable models were created using minimum Bayesian information criteria to generate a final model including only significant (*P* < 0.05) effects.

## Results

In total, 1,925 examination items were evaluated. Examination items were authored by 50 faculty members and appeared on 46 examinations in 16 third year courses (12 core and four elective courses) representing 39 credit hours (33 core and six elective credits); 1689 questions were multiple choice items and 236 were true/false items.

### Item-writing flaws

Approximately 28.8 % of examination items (*n* = 554) were identified as free of item-writing flaws. One item-writing flaw was noted in 33.9 % of the questions, and 37.3 % were identified to have more than one item-writing flaw (two flaws = 384 items, three flaws = 201 items, four flaws = 90 items, five flaws = 30 items, six flaws = 13 items, seven flaws = 1 item). The frequency of specific item-writing flaws appears in Table 1.

**Table 1:** Frequency of item-writing flaws

Awkward stem structure	494	29.4 %
Implausible distractors	386	22.9 %
Longest response is correct	347	20.6 %
True-false distractors	288	17.1 %
Grammatical Clues	259	15.4 %
Negative stem	198	11.8 %
Vague language	188	11.2 %
Unfocused question	147	8.7 %
Absolute terms	99	5.9 %
Misleading stem	76	4.5 %
Mutually-exclusive distractors	53	3.6 %
All of the above	40	2.4 %
None of the above	28	1.7 %
Complex or K-type	15	0.9 %

(*n* = 1682 multiple choice items)

Some items contained more than one flaw

### Distractors

Of 1689 multiple choice items, 19 questions had two distractors, 986 questions had three distracters, and 684 questions had four distractors. The presence of three and four distractors did not impact the mean (± standard error) discrimination index (0.182 ± 0.04 and 0.186 ± 0.08, respectively). Author bias was not detected in the placement of correct response options with four response options (A – 25.1 %, B – 25.8 %, C - 23.9 %, D – 25.1 %) or five response options (A – 20.2 %, B – 21.5 %, C – 19.2 %, D - 20.0 %, E - 19.3 %).

### True/false items

Of 236 true/false items, 111 were true (true was the correct response) and 126 were false statements (false was the correct response). The mean (± standard error) percent correct of true statement items was higher (95.2 % ± 6.7) and the mean discrimination index was lower (0.068 ± 0.05) than the mean percent correct (92.3 % ± 7.9) and discrimination index (0.108 ± 0.080) for false statement examination items.

### Item complexity

More than half of all examination items (61.6 %) were considered lower level recall questions with 401 items (20.8 %) categorized as level I factual recall and 785 items (40.8 %) categorized as level II conceptual recall. Six hundred and four questions (31.4 %) required direct application of knowledge in a simple scenario (Level III) and 133 examination items (6.9 %) required students to analyze conceptual knowledge through interpretation of visual aids and/or complex case material (Level IV). Two examination items asked students to evaluate procedural knowledge through the presentation of a case vignette (Level V). These two items had the longest stem length in this series of 1925 items (12 and 17 lines), highlighting the limitation of this question type.

Interpretation of ancillary materials within examination items was uncommon (12 %). Only 123 examination items (6.4 %) required interpretation of a visual aid; gross or histologic images were most common (*n* = 81), followed by line drawings or ECG tracings (*n* = 22), radiographic or ultrasonographic images (*n* = 14), and video recordings (*n* = 6). Forty-nine examination questions required the examinee to perform a calculation, and 60 questions required students to interpret four or more laboratory values. Six hundred and seventy three examination items (35 %) were classified as case-based questions. The majority of case-based questions (80.1 %) and items requiring interpretation of ancillary materials (72.2 %) were classified at Level III complexity or higher.

**Fig. 1:**
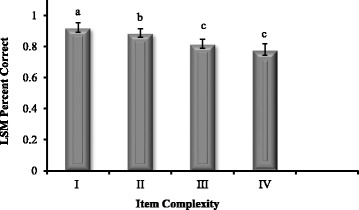
Relationship between item complexity (cognitive level I-IV) and item difficulty (percent correct). As question complexity increased (cognitive level I-IV), the percentage of correct responses decreased. (Letters indicate statistical *p* < 0.028 differences among levels of item complexity)

**Fig. 2:**
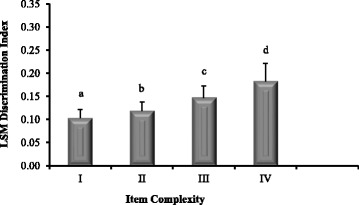
Relationship between item complexity and item discrimination index. As item complexity increased (cognitive level I-IV), the item discrimination index also increased. (Letters indicate statistical *p* < 0.028 differences among levels of item complexity)

For the entire data set, the mean (± standard error) percent correct was 83.3 % (± 17.5) and the mean discrimination index was 0.18 (± 0.17). As question complexity increased (cognitive level I-IV), the percentage of correct responses decreased (Fig. 1). The presence of an implausible distractor resulted in easier examination items (95 % +/ −4.2 correct) compared to questions without an implausible distractor (80.1 % +/ −4.0 correct). These same two variables were also associated with discrimination index values. Mean discrimination index values and question complexity were positively associated (Fig. 2). Examination items with implausible distractors were associated with lower discrimination values (0.09; 95 % CI: 0.06 to 0.12) than questions without implausible distractors (0.20; 95 % CI: 0.14–0.28).

### Difficult, discriminating questions

**Fig. 3:**
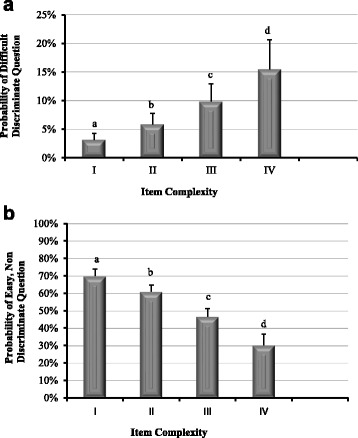
**a**. Item complexity and probability of creating a difficult, discriminating question. The probability of creating a difficult, discriminating question (< 80 % correct; > 0.20 discrimination index) increased with increasing item complexity (cognitive level I-IV). (Letters indicate statistical *p* < 0.028 differences among levels of item complexity). **b**. Item complexity and probability of creating an easy and non-discriminating question. The probability of creating an easy and non-discriminating question (> 90 % correct; <0.15 discrimination index) decreased with increasing item complexity (cognitive level I-IV). (Letters indicate statistical *p* < 0.028 differences among levels of item complexity)

Approximately 30.2 % (*n* = 573) of examination items were classified as difficult, discriminating questions (discrimination index >0.20; percent correct <0.85). Multivariable analysis revealed four question characteristics were associated with the likelihood of creating difficult, discriminating examination items: item complexity, series of true/false distracters, implausible distractors, and all of the above. Not surprisingly, as item complexity increased, the likelihood of creating a discriminatory question increased (Fig. 3a). The probability of writing discriminating, difficult questions decreased when implausible distractors and “all of the above” were used, and increased if the distractors were comprised of a series of true/false questions (Table 2).

**Table 2:** Probabilities of item discrimination and difficulty based on item-writing flaws

Probability (± SE) of creating a discriminating, difficult question (< 80 % correct; > 0.20 discrimination index).		
	NO	YES
Implausible distractors	27.11 % ±8.31	1.83 % ±0.95
Use of *all of the Above*	13.78 % ±4.23	4.15 % ±2.61
Series of true/false distractors	6.16 % ±2.58	9.54 % ±4.05
Probability (± SE) of creating a poorly discriminating, easy question (> 90 % correct; < 0.15 discrimination index).		
	NO	YES
Implausible distractor	31.66 % ±7.05	81.42 % ±5.35
Series of true/false distractors	64.56 % ±7.38	52.71 % ±9

Some items contained more than one flaw

### Poorly discriminating, easy questions

Approximately 43.4 % (*n* = 824) of examination items were categorized as poorly discriminating, easy questions (discrimination index <0.15; > 90 % percent correct). Multivariable analysis revealed item complexity, presence of implausible distractors, and series of true false distractors were associated with the likelihood of creating a poorly discriminating, easy question. As question complexity increased (cognitive levels I-IV), the likelihood of creating an easy question decreased (Fig. 3b). Use of implausible distractors was associated with increased probability of poor discrimination, easy questions, whereas asking questions as a series of true-false distractors decreased the likelihood of generating this pattern of item statistics (Table 2).

### Poorly discriminating, difficult questions

Only 3.4 % (*n* = 64) of examination items were categorized as poorly discriminating, difficult questions (discrimination index <0.15; < 70 correct responses). These parameters were selected to identify characteristics of questions that were unproductive in the examination process. Due to the sparse data in this category, statistical models did not converge.

## Discussion

Overall, the mean percent correct (83.3 %) was consistent with target values for examination average in professional education, and the discrimination index (0.18) was disappointingly lower than recommended (0.20) [28, 30]. The frequency of violations of standard guidelines in the current investigation (71.2 %) is similar to or slightly higher than examinations questions prepared by item-writing experts in nursing (46–76.7 %), accounting (75 %), and medical education (46–67.7 %) [9, 13, 31–35]. The most common item-writing flaws were awkward stem structure, implausible distractors, and longest response is correct. In medical education, commonly reported item-writing flaws include unfocused questions, negatively-worded stems, implausible distractors, all of the above, none of the above, and K-type item formats [9, 13, 32]. In the current study, negative stem and unfocused questions were observed with moderate frequency (< 10 % of items), and use of all of the above, none of the above, and K-type questions was rare. Inappropriate use of implausible distractors is commonly reported from many educational venues.

### Item-writing flaws and discrimination value

Examination items with implausible distractors were less able to discriminate prepared from unprepared students (DI ~0.09), and were easier for all students (~95 % correct) than other examination items. Not surprisingly, the probability of writing challenging, yet discriminating questions decreased when implausible distractors were used. The negative impact of implausible distractors on indices of item quality is a recurring theme in medical education [28].

Implausible distractors are often used as filler, because it is difficult to develop three or four plausible (functional) distractors [29]. Instructors are advised to avoid padding examination items with implausible distractors merely to ensure the same number of response options in each question [29]. Examination uniformity is artificial, and only serves to lengthen the examination without improving the quality or discrimination value. Numerous reports (including the present study) have demonstrated similar item difficulty, discrimination value, and test score reliability between examination items with three and four distractors [2, 16, 18, 29, 36]. Faculty can evaluate post-examination analysis to identify functional and implausible (<5 % selection) distractors; implausible distractors should be eliminated and/or rewritten for subsequent examinations [28, 29].

Examination items with a series of true/false response options were identified more frequently (~11 %) in these data than reported by others (2.8 %) in medical education [13]. The current study found this question format to increase the probability of writing discriminating, difficult questions. Based on a favorable discrimination index, this question type did not appear to disadvantage prepared professional students. Nonetheless, examination-writing experts caution against the use of this format, and despite these results, examination writers should be aware that use of this item format may disadvantage students.

### Question complexity and discrimination value

Higher cognition items require students to assimilate facts, apply knowledge, and predict outcomes, and are more discriminating than factual recall. As question complexity increased (cognitive level I through IV), examination items became more challenging (lower percent correct) and more discriminating; higher question complexity was identified as a feature of difficult, discriminating questions. Medical professionals are expected to use clinical findings and diagnostic results to make decisions that guide treatment planning and prognosis [3, 6, 26]. Ideally, didactic course examinations should be designed to stimulate and evaluate student ability to assimilate and apply complex information (Levels III-V) [1, 3, 32]. Challenging examination items that require higher cognitive skills are positively correlated to content retention and student preparation for subsequent examinations, termed test-enhanced learning [37]. Unfortunately, examination items testing factual recall (20.8 %) and conceptual recall (40.8 %) were common in this survey of examination items targeted for third year veterinary students. In other medical educational settings, the percentage of factual and conceptual recall items is even higher (70–100 %) [9, 13, 38]. In general, instructor-generated questions and examination items targeting third year medical students have a higher percentage of items requiring higher cognitive levels (up to 28.3 %) [9, 13, 38]. The current study is comprised of instructor-generated questions intended for third year students, and appears to have the highest proportion of examination items requiring application and higher cognition (Levels III/IV: 38.4 %) among the cited literature with similar-sized data sets. Despite the relatively high incidence of Level III and IV questions in this study compared to other published reports, the ideal overall percentage for student approaching clinical training should be greater than 60 % [1].

Item-writing experts concede it is difficult and time-consuming to develop multiple-choice items that measure higher cognitive skills [10, 11, 14, 27, 29, 38]. Case-based questions and questions requiring interpretation of diagnostic testing provide a natural format for creating examination items that assess higher cognitive skills. In these data, 35 % of examination items were classified as case-based questions and 12 % required interpretation of ancillary materials (image, video, > 4 laboratory values, calculation), and the majority of these questions were classified at cognitive level III or higher. Interpretation of a radiographic image, video recording, or complex laboratory results is a natural strategy to strengthen item complexity and develop decision-making skills in students. The number of questions requiring calculation in the data set (2.5 %) was particularly disappointing. Applied math skills have been identified as a weakness among clinical students and one course coordinator (clinical pharmacology) requires each instructor to include an applied math problem for the final examination. Most examination items requiring a calculation originated from the clinical pharmacology course.

In the current study, the discrimination index of true/false questions was poor overall. However, false statements were slightly more discriminating than true statements. Discriminating true/false examination items are difficult to construct. Student guessing negatively impacts the discrimination value of true/false questions. Historically, false statements are slightly more discriminating than true statements, and experts recommend test-writers provide slightly more false statements than true statements for that reason [19, 20]. As recommended, CVM faculty posed slightly more false statements in their true/false question sets.

### Positioning the correct response

If unsure of the correct response, students are coached to select option C and avoid option E to increase their chance of obtaining a higher score. In the current study, correct answers were distributed across all options (A, B, C, D, and E); no author bias was noted in the placement of correct options. Most CVM faculty members are aware of these patterns and randomize response options alphabetically or numerically. Examination authors are encouraged to evenly distribute correct responses throughout all response options.

### Limitations

There are two major limitations of this investigation. One is the source of analyzed examination items. All examination items were written by clinical faculty, intended to test information delivered via didactic course work during the third year of the veterinary curriculum from a single institution. The results may not directly extrapolate to course work in basic sciences, other veterinary institutions, or other medical disciplines. The second is the interpretation of Bloom’s taxonomy. Other investigations in clinical medicine employed alternative modifications of Bloom’s taxonomy with fewer categories [39]. Extrapolation of these results may not translate directly to a three-level system. Additionally, curriculum experts may rate multiple choice items at lower levels of complexity than individuals with content expertise [39]. Determination of cognitive level was made by two raters (BRR and DCR) with expertise in clinical medicine.

## Conclusions

Although many item-writing flaws identified in this study did not impact the indices of difficulty or discrimination value, standard item-writing guidelines should be followed to improve the clarity and consistency of examination items. Item-writing flaws identified as disruptive to indices of performance for professional students include implausible distractors, use of “all of the above”, and series of true/false response options. Faculty training should place particular emphasis on avoiding these item-writing flaws.

Higher question complexity (cognitive level III through IV) was identified as a feature of discriminating examination questions. Examination items that require higher cognitive skills are correlated to student learning and development, particularly in preparation for clinical training [1, 3, 26, 27, 38]. Clinically-applied course content lends itself to case-based examination items, which provide a natural platform for construction of examination items requiring higher cognitive skills. Clinical faculty delivering didactic course material are encouraged to develop case vignette-based multiple choice examination materials.

## Additional file

Additional file 1: Examples of select item-writing flaws. (PDF 244 kb)

## Abbreviations

CVM: College of veterinary medicine
NBME: National Board of Medical Examiners

## Footnotes

1 OpScan® 6 OMR Scanner. SCANTRON Corporation. 1313 Lone Oak Rd., Eagan, MN 55121
