# Supplementary material for: The impact of item-writing flaws and item complexity on examination item difficulty and discrimination value
Source: BMC Med Educ. 2016 Sep 29;16:250. doi: 10.1186/s12909-016-0773-3 (PMC5041405; doi:10.1186/s12909-016-0773-3)
Supplement: Additional file 1: — Examples of select item-writing flaws. (PDF 244 kb) [file 12909_2016_773_MOESM1_ESM.pdf]

Additional file 1. Examples of select item-writing flaws.

Longest response is correct.

Stem: Which of the following is the most important clinical consideration with ketamine administration in cats?

a. Hepatic metabolism is the primary route of elimination and should be avoided in cats with hepatic disease.
b. IM ketamine, when used as a premedication, may produce an excessive depth of anesthesia when cats are subsequently exposed to induction and inhalation agents.
c. Ketamine induction can produce Cheyne-Stokes breathing in cats.
d. Ketamine causes profound hypotension in cats.

Grammatical clues or inconsistencies between stem and distractors.
Mutually exclusive distractors.

Stem: Which of the following is consistent with AAEP guidelines for vaccination of adult horses?

a. Juveniles are vaccinated against influenza starting at 6 months of age. (Not an adult.)
b. Serous nasal discharge may be observed after intranasal *Streptococcus equi* vaccination. (Not a guideline.)
c. Core vaccine guidelines protect from diseases acquired without horse to horse contact (e.g rabies, tetanus). ("Guideline" appears in stem and response.)
d. Horses are protected from common contagious diseases (e.g. equine herpesvirus, influenza) using core vaccines. (Options c and d are mutually exclusive.)

Implausible distractors
Response options are a series of true/false questions.

Stem: Select the correct statement regarding the benefit of aquatic therapy for rehabilitation of musculoskeletal injury in dogs.

a. Decreases forces on the joints due to decreased buoyancy. (implausible; 1 student selected)
b. Decreases inflammation due to decreased temperature of water.
c. Increases strength due to resistance to moving through water.
d. Increases owner compliance due to high cost. (implausible; 0 students selected)

Awkward stem structure. (Finish the sentence, fill in the blank)
Longest response is correct.

Stem: Hypoxic vasoconstriction is ________________.

a. Associated with pulmonary vasodilation to pulmonary regions with atelectasis.
b. A physiologic phenomenon that redirects pulmonary blood flow to alveoli with higher oxygen content. (Structure differs from other options.)
c. Provides valuable compensatory mechanism in horses with chronic heaves. (Syntax error between stem and distractor.)
d. Prevents high altitude disease in cattle. (Syntax error between stem and distractor.)

Stem: In healthy fasted horses, the ___________ and ___________ bilirubin are elevated, and the ___________ and ___________ bilirubin are unaffected.

a. **Indirect and total; BUN and direct**
b. Direct and total; albumin and indirect
c. GGT and indirect; direct and total
d. ALP and total; indirect and glucose

Extraneous or misleading information in the stem (underlined).

Stem: You recently provided care for a 2-yr old Thoroughbred racehorse in training with serous nasal discharge, cough, and fever of 2 days duration. Nasal secretions were positive on influenza A ELISA test. The colt has been symptom-free for one week. The owner worries the colt is gaining weight, losing condition, and becoming rambunctious. "He is going to injure himself in the stall." He has an important race at Santa Anita in three weeks, and needs to get back in training. When should this horse be put back into training?

Stem: You are presented with a 2-yr old Labrador with an acute, non-weight-bearing lameness of the right hind limb. The owner reports that the dog ran off while training for a field trial the previous evening and returned in the early hours of the morning. It was a dark and stormy night in May. After eliciting an abnormal thumb test and triangle test, you are able to make a diagnosis. Only last week you performed pelvic radiographs on this dog to submit for OFA certification – the hips looked very good. Which of the following diagnostic tests is indicated in this dog?

Unfocused question. (Distractors are unrelated or distantly related.)
Response options are a series of true/false questions.

Stem: Pick the correct response.

a. **C-section with OHE generally requires more assistants versus hysterotomy.**
b. C-section with OHE has a lower survival rate for pups versus hysterotomy.
c. In cats, 35-50% of mammary tumors are malignant.
d. Pyometras in dogs occur during proestrus.

(Surgery, prognosis, pathophysiology of reproductive organs.)

Stem: Pick the correct statement regarding neurologic conditions in horses.

a. West Nile virus is spread horse to horse through close contact.
b. SAG 1 ELISA on CSF is the most reliable test for equine protozoal myelitis.
c. Horses maintain a functional menace response with cortical blindness.
d. **Treatment of tetanus requires administration of antitoxin and toxoid.**

(Four diseases and four objectives - transmission, treatment, clinical signs, diagnostic testing.)
